# Cost-effectiveness evaluation of add-on dapagliflozin for heart failure with reduced ejection fraction from perspective of healthcare systems in Asia–Pacific region

**DOI:** 10.1186/s12933-021-01387-3

**Published:** 2021-10-09

**Authors:** Chia-Te Liao, Chun-Ting Yang, Han Siong Toh, Wei-Ting Chang, Hung-Yu Chang, Fang-Hsiu Kuo, Mei-Chuan Lee, Yi-Ming Hua, Hsin-Ju Tang, Carol Strong, Huang-Tz Ou

**Affiliations:** 1 Department of Public Health, College of Medicine, National Cheng Kung University, Tainan, Taiwan; 2 Division of Cardiology, Department of Internal Medicine, Chi Mei Medical Center, Tainan, Taiwan; 3 Department of Electrical Engineering, Southern Taiwan University of Science and Technology, Tainan, Taiwan; 4 Institute of Clinical Pharmacy and Pharmaceutical Sciences, College of Medicine, National Cheng Kung University, Tainan, Taiwan; 5 Department of Intensive Care Medicine, Chi Mei Medical Center, Tainan, Taiwan; 6 Department of Health and Nutrition, Chia Nan University of Pharmacy & Science, Tainan, Taiwan; 7 Department of Biotechnology, Southern Taiwan University of Science and Technology, Tainan, Taiwan; 8 Heart Center, Cheng Hsin General Hospital, Taipei, Taiwan; 9 Faculty of Medicine, School of Medicine, National Yang Ming Chiao Tung University, Taipei, Taiwan; 10 Department of Pharmacy, Chi Mei Medical Center, Tainan, Taiwan; 11 Department of Nursing, Chang Gung University of Science and Technology, Chiayi, Taiwan; 12 Department of Pharmacy, College of Medicine, National Cheng Kung University, Tainan, Taiwan; 13 Present Address: Division of Cardiology, Department of Internal Medicine, Chi Mei Medical Center, Tainan, Taiwan

**Keywords:** Heart failure, Dapagliflozin, Cost-effectiveness

## Abstract

**Background:**

With emerging evidence on the efficacy of adding dapagliflozin to standard care for patients with heart failure with reduced ejection fraction (HFrEF), this study assessed the cost-effectiveness of add-on dapagliflozin to standard care versus standard care alone for HFrEF from the perspective of healthcare systems in the Asia–Pacific region.

**Methods:**

A Markov model was applied to project the outcomes of treatment in terms of lifetime medical cost and quality-adjusted life-years. The transition probabilities between health states in the model were obtained from the Dapagliflozin in Patients with Heart Failure and Reduced Ejection Fraction trial. Country-specific costs and utilities were extracted for modeling. The incremental cost-effectiveness ratio against a country-specific willingness-to-pay threshold was applied to determine the cost-effectiveness of treatment. A series of sensitivity analyses were performed to ensure the robustness of the study results. Costs are presented in 2020 United States dollars.

**Results:**

The incremental cost-effectiveness ratios for add-on dapagliflozin versus standard care alone were $5277, $9980, $12,305, $16,705, and $23,227 per quality-adjusted life-year gained in Korea, Australia, Taiwan, Japan, and Singapore, respectively. When using add-on dapagliflozin to standard care versus standard care alone, ~ 100% of simulations were cost-effective at a willingness-to-pay threshold of one gross domestic product per capita of the given Asia–Pacific country; however, the probability of being cost-effective for using add-on dapagliflozin decreased when the time horizon for simulation was restricted to 18 months and when the cardiovascular mortality for the two treatments (43.8% and 33.0%, respectively) was assumed to be the same. The cost-effectiveness results were most sensitive to cardiovascular mortality of treatment.

**Conclusions:**

Adding dapagliflozin to standard care is cost-effective for HFrEF in healthcare systems in the Asia–Pacific region, which supports the rational use of dapagliflozin for HFrEF in this region.

**Supplementary Information:**

The online version contains supplementary material available at 10.1186/s12933-021-01387-3.

## Background

The prevalence and incidence of heart failure (HF) have increased along with population aging. The disease and economic burdens caused by HF are a major challenge for healthcare systems [1, 2]. The prevalence of HF in Asia (1.3–6.7%) is higher than that in Western countries (about 1–2%) [1]. The global economic burden attributable to HF has been estimated to be US$108 billion per annum, of which direct costs (e.g., healthcare services and medications) and indirect costs (e.g., loss of productivity caused by morbidity and mortality) accounted for around 60% and 40%, respectively [2]. Hospitalization for HF (HHF), one of the most major clinical problems among patients with HF, has been reported to be a key driver of HF-associated medical expenditure in several studies [3–5].

To ameliorate the substantial burden to individuals with HF, caregivers, and the society, the primary goals of HF management are to alleviate clinical symptoms, enhance quality of life, prevent hospital admission, and reduce patient mortality [6]. Evidence-based pharmacological therapies, including renin–angiotensin–aldosterone system agents, beta blockers, mineralocorticoid receptor agonists, and ivabradine, are mostly limited to patients with HF with reduced ejection fraction (HFrEF) rather than those with HF with preserved ejection fraction. Nonetheless, more cost-effective treatments are desirable.

Sodium-glucose cotransporter-2 inhibitors (SGLT-2is), which are currently used as glucose-lowering agents with well-known clinical and economic value for patients with type 2 diabetes [7–9], have recently been shown to have promising efficacy in lowering HHF and cardiovascular death among the HF population regardless of the diabetes status of patients. Although the use of dapagliflozin, an SGLT-2i, for patients with HF with preserved ejection fraction remains controversial, the Dapagliflozin in Patients with Heart Failure and Reduced Ejection Fraction (DAPA-HF) trial has revealed that dapagliflozin added to standard care versus standard care alone yielded significant 30% and 18% reductions in HHF risk and cardiovascular mortality in patients with HFrEF, respectively [10]. In addition, the cost-effectiveness of add-on dapagliflozin has been reported in some countries [11–15]. Nonetheless, cost-effectiveness may depend on the specific healthcare system. A cost-effectiveness evaluation of adding dapagliflozin to standard care for HF populations has not been conducted for most Asia–Pacific countries.

Against this background, the aim of the present study was to assess the cost-effectiveness of add-on dapagliflozin to standard care in patients with HFrEF from the perspective of healthcare systems in the Asia–Pacific region.

## Methods

### Model structure

**Fig. 1:**
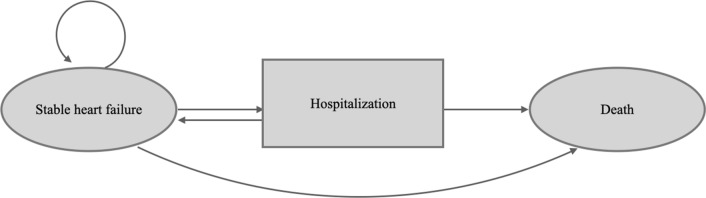
Overview of model structure for base-case cost-effectiveness analysis

A Markov model with discrete health states was used to simulate the disease progression of patients with HFrEF. The model was constructed according to three major health states, namely stable HF or post-HHF, acute HF events (i.e., emergency visit or HHF due to worsening HF), and death (Fig. 1). In the base-case analysis, it was assumed that all patients had started treatment at 66 years old and progressed from stable HFrEF without acute events through a Markov model with monthly cycles until death or a 15-year horizon was reached, which is close to the life expectancy in Taiwan (81.3 years in 2020 [16]). Cost and utility data were discounted using an annual discounting rate of 3% according to Taiwan’s Guidelines of Methodological Standards for Pharmacoeconomic Evaluation [17]. Details of the model structure and input parameters in the base-case analysis are provided in Additional file 1 and Table 1, respectively.

**Table 1:** Input parameters for base-case cost-effectiveness analysis of add-on dapagliflozin to standard care versus standard care alone for heart failure

Monthly transition probabilities^a^	Estimate	Standard deviation	Distribution	Data source
Hospitalization for HF				
Add-on dapagliflozin	0.005611395	0.001533432	Beta	DAPA-HF trial [10]
Standard care	0.007881397	0.001816007	Beta	
Cardiovascular death				
Add-on dapagliflozin	0.005509456	0.001519518	Beta	
Standard care	0.006698743	0.001675218	Beta	
Non-cardiovascular death				
Add-on dapagliflozin	0.005509456	0.001519518	Beta	
Standard care	0.001312441	0.000743514	Beta	

Cost parameters (per month)	Cost (US$)	Range	Distribution	Data source
Drug costs of dapagliflozin (10 mg once daily)	28.6	± 14.3	Gamma	NHIRD
Stable HF	450	± 225	Gamma	
Emergency visit or hospitalization for HF	2887	± 1443.5	Gamma	
Cost during month before cardiovascular death	3430	± 1715	Gamma	
Cost during month before non-cardiovascular death	3390	± 1695	Gamma	

Health utilities parameters	Estimate	Standard error	Distribution	Data source
Stable HF	0.770	0.016	Beta	[19]
Utility decrement associated with aging (per year increase)	− 0.0016	0.0001	Beta	
Utility decrement of emergency visit or hospitalization related to HF	− 0.321	0.02	Beta	

*DAPA-HF* dapagliflozin in patients with heart failure and reduced ejection fraction, *HF* heart failure, *NHIRD* National Health Insurance Research Database

^a^The monthly transition probabilities were transformed as follows: (1) Probability (obtained from DAPA-HF trial) transformed to a rate: [− ln (1 − p)]/t. (2) Rate transformed to a probability (monthly transition probability applied in the analyses): 1 − exp(– rt), where r is the rate, p is the probability, and t is the time

### Modeled population

A hypothetical cohort that reflected the patient population in the DAPA-HF trial [10] was simulated. Briefly, the eligibility criteria of patients enrolled in the DAPA-HF trial included an age of ≥ 18 years, ejection fraction of ≤ 40%, and New York Heart Association functional class (NYHA Fc) II, III, or IV symptoms. All patients were required to receive standard HF care, including device therapy (i.e., implantable cardiovertier-defibrillator, cardiac resynchronization therapy) and drug therapy (i.e., angiotensin-converting-enzyme inhibitors/angiotensin-receptor blockers/sacubitril-valsartan, and beta blockers). Patients with side effects to SGLT-2is, type 1 diabetes, symptoms of hypotension or a systolic blood pressure of < 95 mmHg, or an estimated glomerular filtration rate of < 30 ml/min/1.73 m^2^ were excluded. The detailed inclusion and exclusion criteria for the DAPA-HF trial subjects and the baseline characteristics of the patients are presented elsewhere [10]. No differences in patient baseline characteristics between the treatment groups in the DAPA-HF trial were reported [10].

### Model assumptions and transition probabilities

All patients using dapagliflozin 10 mg daily added-on to standard care (add-on dapagliflozin group) or standard care alone (standard care group) without any clinical events entered the state of stable HF at the first cycle. Next, the patients progressed to the state of acute HF events or death based on the given transition probabilities between health states. The monthly transition probabilities were derived from the results reported in the DAPA-HF trial [10]. The annual probabilities of given events (e.g., HHF) measured as the annual number of events over a median follow-up of 18.2 months in the DAPA-HF trial were first obtained. Then, the monthly transition probabilities were converted from the annual probabilities of given events. A detailed formula for the above estimations and a full list of monthly transition probabilities are provided in Table 1.

### Cost and utility estimates

The healthcare costs of HF management were measured from the nationwide claims-based database, the National Health Insurance Research Database (NHIRD), in Taiwan. Patients with stable HF were identified using the following criteria: at least two HF diagnoses (International Classification Diseases, Ninth Revision, Clinical Modification: 428) in the ambulatory care department within 180 days in 2015 and no hospital admissions or emergency visits due to HF in the 180 days preceding the first HF diagnosis in 2015. Among these patients, the cost for chronic HF care was calculated as the sum of medical costs divided by the total follow-up period, which started from the first HF diagnosis until the occurrence of an acute HF event (i.e., emergency visit or HHF), death, or the end of December 2018, whichever came first. To estimate the cost of acute HF events, patients with an HF event were first identified, and then the medical costs in the first and following months were measured separately as the event-month and the state-month costs for HF events, respectively. The event-month costs were calculated as the costs in the first month of an acute event and the state-month costs were defined as the costs in the second and subsequent months of the event. Regarding the medical costs associated with cardiovascular and non-cardiovascular deaths, the death status and the cause of death of each patient were ascertained from the Cause of Death files in the NHIRD, and the medical costs for the month before death were estimated. The cost data were adjusted to 2020 prices using the medical component of the consumer price index in Taiwan [18] and are presented in United States dollars (US$).

The utility scores, which ranged from 0 to 1, for each health state were obtained from published literature with consideration of the appropriateness of the study population (e.g., race/ethnicity, disease population), and the quality and comprehensiveness of the source [19]. The patients were assumed to have a baseline utility score of 0.77 at the first cycle in the model (i.e., stable HF state). Decrements in utility scores associated with HHF and aging were applied to the following cycles. Detailed cost and utility inputs are shown in Table 1.

### Base-case cost-effectiveness analysis

The lifetime medical cost was the sum of the medical costs in all cycles (for each cycle, the medical cost was estimated by multiplying the medical cost of each health state by the number of patients in the given health state) over a 15-year projection. Similarly, the total quality-adjusted life-years (QALYs) over 15 years were the sum of the QALYs in all cycles (for each cycle, QALYs were estimated by multiplying the utility value associated with each health status by the number of patients in the given health state). To evaluate the cost-effectiveness of add-on dapagliflozin versus standard care alone, the incremental cost-effectiveness ratio (ICER), calculated as the incremental lifetime medical cost divided by the incremental QALYs between the two treatments, are presented. Further, we applied the willingness-to-pay (WTP) thresholds of US$ 25,000 and US$ 75,000 per QALY gained, which are close to the one and three gross domestic product (GDP) per capita of Taiwan in 2020, respectively, to determine whether add-on dapagliflozin versus standard care is very cost-effective (i.e., ICER ≤ US$ 25,000) and cost-effective (i.e., ICER ≤ US$ 75,000).

### Sensitivity analyses

Deterministic sensitivity analyses (DSAs) and probabilistic sensitivity analyses (PSAs) were conducted to quantify the variations in the estimation of ICER caused by parameter uncertainties. In the DSAs, the lower and upper bounds of each model input were applied to assess the dominant parameters in the ICER estimation. The PSAs, which allowed all model inputs to be simultaneously varied in the given ranges and followed predefined distributions (Table 1), were performed using Monte Carlo simulation with 10,000 iterations.

### Subgroup and scenario analyses

Analyses were further stratified by age (≤ 65 or > 65 years), race (Black, Asian, and White), type 2 diabetes status, cause of HF (ischemic or non-ischemic heart failure), NYHA Fc (II, or III/IV), and baseline left ventricular ejection fraction level (> or ≤ median value) to assess whether the study results differed according to patients’ underlying conditions. Additionally, several scenario analyses were conducted to confirm the robustness of the study findings. First, the simulation horizons were extended to 30 years and restricted to 18 months which matched the follow-up period of the DAPA-HF trial [10], to assess the uncertainty that arose from the length of the simulation period. Second, the discounting rates were set at 0% and 10%. Third, the trial included a selective patient population (e.g., patients with a high risk for HF progression and optimal adherence to treatment) and thus its treatment efficacy results may not be generalizable to real-world settings. In this regard, a scenario analysis was conducted where the efficacy measures of add-on dapagliflozin (i.e., the risk of cardiovascular death, non-cardiovascular death, and hospitalization or emergency visit for heart failure) were assumed to be equal to those of standard care to account for the uncertainty of applying trial results into real-world practice. This was done to examine whether our study findings remained robust when the treatment benefit was not revealed in real-world settings. Fourth, although the DAPA-HF trial [10] revealed a non-significant difference in the occurrences of adverse events between the two treatment arms, several health states associated with hospitalizations attributable to the adverse events of treatments were further considered in the simulation model for a more comprehensive evaluation of the effects of add-on dapagliflozin versus standard care (Additional file 2). The model inputs of the considered adverse events are presented in Additional file 3. Fifth, we adjusted the monthly cost of dapagliflozin within a certain range to reflect the variations in drug acquisition costs under different healthcare systems or due to changes in the reimbursement policies of Taiwan’s National Health Insurance. Lastly, because the results of cost-effectiveness evaluation are likely to be country- or ethnicity-specific, we applied these analyses to other developed countries with similar life expectancy and universal national health insurance coverage in the Asia–Pacific region, namely Japan, Korea, Singapore, and Australia, to evaluate the cost-effectiveness for these countries (detailed model inputs are available in Additional file 4).

All of the above-mentioned analyses were conducted using SAS 9.4 and TreeAge Pro software.

## Results

### Base-case analysis

In the 15-year simulation, 590 and 753 HHF events occurred in the add-on dapagliflozin and standard care groups, respectively. Approximately 70% of the study patients died at the end of the simulation (69.9% in the add-on dapagliflozin group and 76.5% in the standard care group). Compared with standard care alone, adding on dapagliflozin yielded an additional 1.25 life-years (LYs) (14.71 versus 13.46) and 0.94 QALYs (11.03 versus 10.09), with an incremental medical cost of US$ 11,304 (Table 2). The ICER values were US$ 9,080 per LY gained and US$ 12,305 per QALY gained.

**Table 2:** Cost-effectiveness of add-on dapagliflozin to standard care versus standard care alone in the base-case, subgroup, and scenario analyses, from the perspective of healthcare system in Taiwan

	Cost (US$)			QALYs or LYs			ICER (US$)	Probability of cost-effectiveness, dapagliflozin vs. standard care (%)	
	Add-on dapagliflozin	Standard care	$$\Delta$$ Costs	Add-on dapagliflozin	Standard care	$$\Delta$$ QALYs or $$\Delta \mathrm{LYs}$$		WTP at US$ 25,000	WTP at US$ 75,000
Base-case analysis									
QALY gain as effectiveness outcome	87,805	76,501	11,304	11.03	10.09	0.94	12,035	99.3	100
LY gain as effectiveness outcome	87,805	76,501	11,304	14.71	13.46	1.25	9,080	100	100
Scenario analyses									
(1) Time horizon									
30 years	104,623	87,940	16,682	13.14	11.60	1.54	10,832	100	100
18 months	16,719	15,732	987	2.10	2.08	0.03	37,386	43.8	61.8
(2) Discounting rate									
0%	104,288	90,112	14,176	13.1	11.89	1.21	11,681	100	100
10%	62,215	55,130	7,085	7.82	7.27	0.54	13,007	97.3	100
(3) Under assumption of equal risk of clinical events between the two treatments									
Cardiovascular death	81,581	76,501	5,081	10.20	10.09	0.11	44,670	33	60.9
Non-cardiovascular death	86,887	76,501	10,387	10.91	10.9	0.82	12,704	98.9	100
Hospitalization for HF	87,995	76,501	11,444	11.03	10.09	0.94	12,204	99.6	100
Emergency visits for HF	87,818	76,501	11,317	11.03	10.09	0.94	12,055	99.9	100
(4) Adverse events of treatments considered in the model	88,074	76,727	11,347	10.95	10.02	0.92	12,288	99.3%	100

*QALY* quality-adjusted life-year, *ICER* incremental cost-effectiveness ratio, *WTP* willingness-to-pay, *LY* life-year, *HF* heart failure

### Sensitivity analyses

**Fig. 2:**
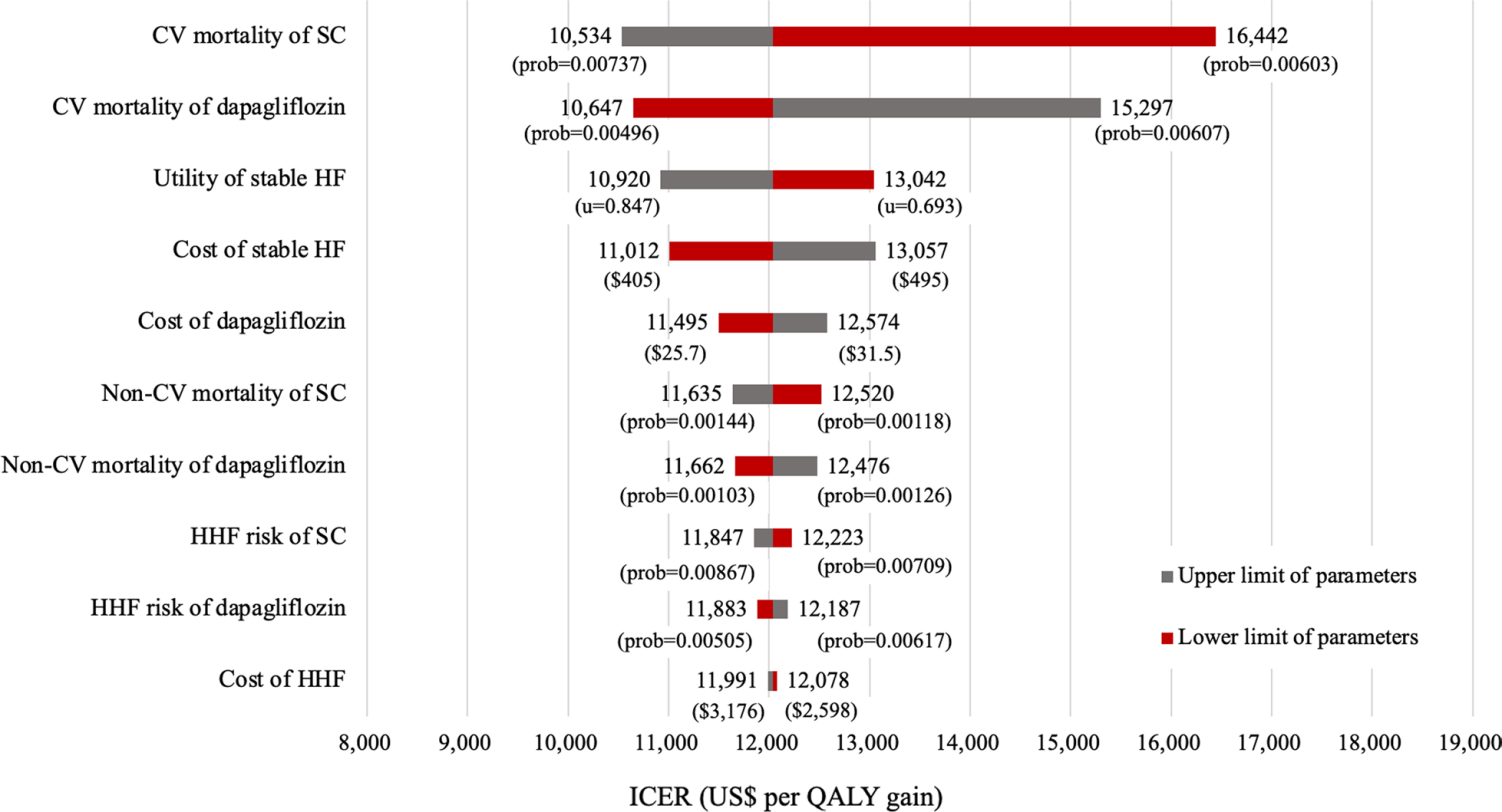
Tornado diagram for results of deterministic sensitivity analysis. *CV* cardiovascular, *SC* standard care, *HF* heart failure, *HHF* hospitalization for heart failure. “prob.” means transitional probabilities, “u” means utility scores, and $ represents the cost in 2020 United States dollars.

Figure 2, a tornado diagram for the DSA results, indicates that the cardiovascular mortalities of the standard care and add-on dapagliflozin group were the most dominant parameters affecting the ICER values, followed by the utility of stable HF, cost of stable HF, and the drug acquisition cost of dapagliflozin. As shown in Table 2 and Additional file 5, the PSA results show that 99.3% of simulations were cost-effective at the WTP threshold of US$ 25,000 in Taiwan when dapagliflozin was added to standard care versus standard care alone.

### Subgroup analyses

The subgroup analysis results (Additional file 6) are consistent with the base-case analysis results. Across the subgroups, approximately 100% of simulations in the PSAs were cost-effective when dapagliflozin was used as an add-on therapy to standard care versus standard care alone under the predefined WTP threshold of US$ 25,000 in Taiwan, except for the group of patients with NYHA Fc III or IV.

### Scenario analyses

Table 2 also shows the results of scenario analyses that considered potential uncertainties that arose from the study assumptions and the generalizability of study findings to real-world practice. Under the predefined WTP of US$ 25,000 in Taiwan, the probability of being cost-effective for using add-on dapagliflozin versus standard care alone in the PSAs was above 97% when: (1) the time horizon was extended to 30 years, (2) the discounting rate was set at 0% or 10%, (3) the risks of hospitalization or emergency visit for HF and non-cardiovascular mortality were assumed to be the same for the two treatment arms, or (4) adverse events of treatment were considered to be health states in the model simulation. However, the probabilities decreased to 43.8% and 33% when the time horizon was restricted to 18 months and the same cardiovascular mortality was applied for the two treatment groups, respectively.

Additional file 7 shows the probabilities of being cost-effective for using add-on dapagliflozin versus standard care alone when the monthly drug acquisition costs of dapagliflozin were redefined as 25%, 50%, and 75% of the drug cost in the base-case analyses. The figure shows findings consistent with those of the base-case analyses.

**Fig. 3:**
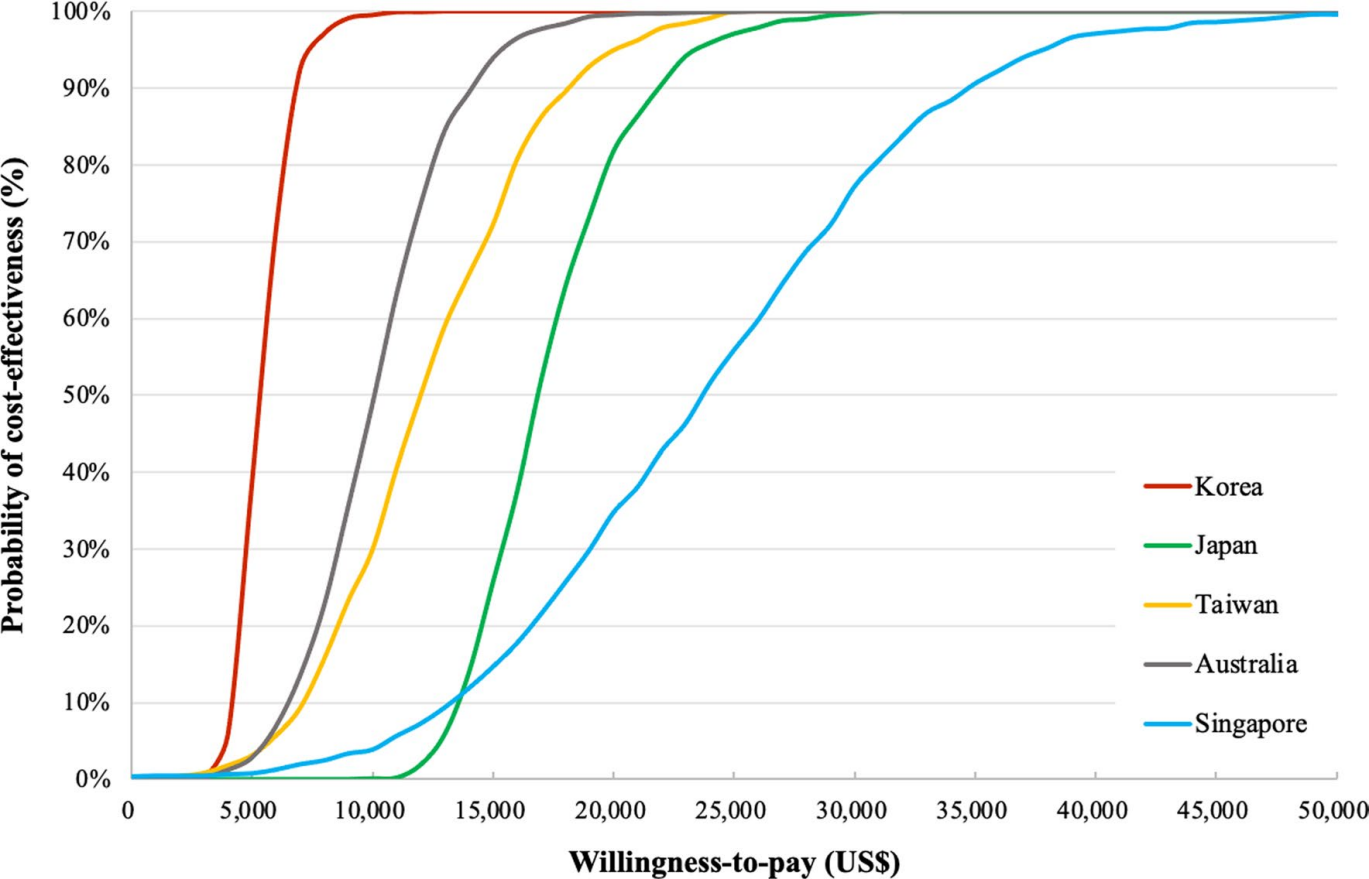
Results of probabilistic sensitivity analyses for cost-effectiveness of add-on dapagliflozin to standard care versus standard care alone in Asia–Pacific countries under different country-specific willingness-to-pay thresholds. GDP per capita (2020): US$ 39,000 for Japan; US$ 30,000 for Korea; US$ 58,000 for Singapore; US$ 52,000 for Australia

In other Asia–Pacific countries (Fig. 3; Additional file 8), when using add-on dapagliflozin to standard care versus standard care alone, approximately 100% of simulations were cost-effective at the willingness-to-pay threshold of one GDP per capita of the given country.

## Discussion

This study provides supporting evidence for adding dapagliflozin to standard care for HFrEF in terms of decreased risks of emergency visits, hospitalization admissions, and deaths associated with HF, and economic benefits from the perspective of healthcare systems in the Asia–Pacific region. The favorable economic result of add-on dapagliflozin is generalizable across Asia–Pacific countries under different cost and utility settings. The robustness of our findings was further confirmed in a series of sensitivity, subgroup, and scenario analyses. Additionally, the cost-effectiveness results under different monthly drug costs of dapagliflozin against different WTP thresholds are provided (Additional file 7), which is of importance for healthcare policy decision-making and for supporting the use of dapagliflozin in HF patients; i.e., for a given WTP threshold of the healthcare system, specific reimbursement costs for dapagliflozin and associated reimbursement policies can be set to achieve cost-effective use of dapagliflozin for HFrEF.

### Comparison of study results with existing evidence

The present study findings are generally consistent with current evidence. McEwan et al. evaluated the cost-effectiveness of dapagliflozin added to standard care versus standard care alone among HF populations in European countries; the ICERs ranged between £5,822 (around US$ 8,112) and €9,406 (around US$ 11,298), and the probability of cost-effectiveness ranged between 91 and 97% under the given WTP thresholds (i.e., £20,000 in UK, and €20,000 in Germany and Spain) [11]. These results are similar to our study results. Given the high medical expenditures and the healthcare system in the US, a previous study found that add-on dapagliflozin versus standard care alone in HFrEF resulted in an additional cost of US$ 83,650 per QALY gained and add-on dapagliflozin was an approximately 89% cost-effectiveness strategy for HFrEF [15]. It was therefore concluded that add-on dapagliflozin in patients with HFrEF had an intermediate economic value.

Regarding the Asia–Pacific region, Savira et al. [13] and Krittayaphong et al. [14] reported that adding dapagliflozin to the standard care of HF was highly cost-effective from the perspective of the healthcare systems in Australia (a 98.8% cost-effectiveness strategy and 0.2% cost-saving strategy) and Thailand (an 87% cost-effectiveness strategy), respectively. However, Yao et al.’s study [12] on a Chinese population with HF showed a relatively low probability of cost-effectiveness (53.1%) for using dapagliflozin as an add-on therapy to standard care. These inconsistent findings may be attributed to differences in study assumptions, input parameters for model simulation, and WTP thresholds across studies. For example, the utility inputs associated with HF health state applied in Yao et al.’s study (i.e., 0.127–0.204) were lower than those in other studies (0.508–0.833) [11–15].

### Dominant parameters for ICER values

According to the DSA results, the cardiovascular mortalities of the standard care and add-on dapagliflozin groups were the first and second most dominant parameters, respectively, that affected the cost-effectiveness results. These results were expected because as shown in the DAPA-HF trial [10], cardiovascular death associated with add-on dapagliflozin versus standard care alone was the most promising efficacy evidence for the difference between these treatments (hazard ratio [95% CI]: 0.82 [0.69–0.98]), and the medical cost of cardiovascular death (i.e., US$ 3,430 for the month of cardiovascular death) was considerably higher compared to other cost inputs in this economic analysis. Although the utility and medical cost inputs of stable HF were also among the top drivers of the ICER variations, their impacts on the ICERs were relatively modest (i.e., changes in these parameters resulted in less than 10% variation in the ICER values). In addition, add-on dapagliflozin versus standard care alone remains cost-effective considering the different costs and utility settings from the perspectives of healthcare systems in other Asia–Pacific countries, including Japan, Korea, Singapore, and Australia. Therefore, as supported by a series of sensitivity and scenario analyses, we believe that our study findings are robust and could be generalizable across Asia–Pacific settings to promote the rational use of dapagliflozin for HFrEF populations in this region.

### Need for real-world evidence of add-on dapagliflozin to standard care for HF

In the scenario analyses where the time horizon was restricted to 18 months and the same cardiovascular mortality was assumed between the two treatments, the probabilities of cost-effectiveness in PSAs for using add-on dapagliflozin versus standard care alone were lower than 50% under the WTP threshold of US$ 25,000 (i.e., one GDP per capita of Taiwan) but were above 60% under the US$ 75,000 (i.e., three GDP per capita of Taiwan) WTP threshold, which still implies a favorable economic benefit of using dapagliflozin as an add-on therapy. These results also suggest that treatment duration and cardiovascular mortality should be considered when evaluating the economic benefit of using dapagliflozin for HF. Moreover, large-scale clinical trials do not support the treatment efficacy of SGLT-2i in HF patients with certain clinical conditions, such as drug-naive patients, patients with severely reduced ejection fraction, and patients with acute decompensated HF [20]. To understand the external validity of these trial results, further studies assessing the effectiveness of adding dapagliflozin to standard care among real-world diverse patient populations are warranted. Such studies will provide more precise estimates for cost-effectiveness analyses that can reflect routine care settings.

Additionally, we found that add-on dapagliflozin showed an 100% cost-effectiveness strategy at a low WTP threshold (i.e., US$ 12,000–US$ 15,000) among patients with NYHA Fc II versus those with NYHA Fc III/IV (Additional file 6d). This implies a significant economic benefit given that dapagliflozin can be timely given or added on to standard care in the early disease stage of HFrEF patients. Future research is encouraged to explore the subset of HFrEF patients who can benefit the most from the use of add-on dapagliflozin to promote personalized medicine and facilitate resource allocation from the perspectives of healthcare providers and reimbursement policymakers, respectively.

### Study limitations

First, the transition probabilities, which were mainly extracted from a randomized controlled trial, may be limited to usual practice. For example, treatment effects between the trial population and real-world patients might be different. We thus performed scenario analyses that assumed non-differential risks of acute HF events, cardiovascular, and non-cardiovascular death between the two treatments; the results showed that add-on dapagliflozin remained cost-effective. Second, considering that the treatment effect may vary across races and healthcare settings, the use of the results from the entire DAPA-HF trial population as the model inputs in this perspective study for the Asia–Pacific region may be of concern. However, evidence on the effect of dapagliflozin use in HF among Asia–Pacific patient populations is limited. Subgroup analyses of Asian patients and Asian regions in the DAPA-HF trial showed consistent results with those of the main analysis, which used all trial patients [10]. Additionally, we performed PSAs considering the wide ranges of model input values that may reflect the treatment effects in different races and a series of subgroup analyses by races (i.e., White, Black, and Asian). The results of these analyses supported the economic benefit of dapagliflozin treatment for HF across different racial groups. Third, the model inputs applied in this study were obtained from multiple sources, which might introduce a certain level of uncertainty to the study results. However, the robustness of our study findings was confirmed by a series of sensitivity and scenario analyses, which minimizes this concern. Fourth, for simplicity, our analyses applied constant transition probabilities over the simulation period despite patient aging; however, the assumption of constant transition probabilities might not reflect the possible changes in the risks of disease progression or death over time with patient aging in the chronic disease course of HF. In the scenario analyses, we thus (1) allowed the transition probabilities to vary within a given range with beta distributions in the PSAs and (2) reiterated the time horizon for a short-term simulation (18 months). The consistent findings between these further analyses and the base-case analyses strengthened our confidence in the study conclusions. Moreover, we plotted the survival curve of a real-world HF population identified from the NHIRD (see Additional file 9) for comparison with the simulated survival outcome of patients estimated from the Markov model (i.e., Fig. 1). The observed and simulated survival curves are comparable, which suggests that the potential impact of disease changes with aging for the modeled population on the lifetime estimation (e.g., LYs) might be negligible. Fifth, indirect costs (e.g., productivity loss due to HHF and HF-related disability) were not analyzed because our primary aim was to evaluate the cost-effectiveness of treatments from the perspective of healthcare payers. Nonetheless, more substantial economic benefits of dapagliflozin use are expected if the costs of productivity loss are included because the add-on dapagliflozin strategy has been reported to significantly reduce the risk of acute HF events and improve the quality of life of patients [10].

Sixth, the model structure in this economic analysis focused on only the cardiovascular outcomes of HF patients. Other clinically important health states, such as kidney diseases, in this population were not considered. However, a valid disease prediction model that comprises the health states of several organ systems (e.g., cardiovascular and kidney systems) in HF patients is lacking, and data on the associated treatment effects, which are important information for transition probabilities in the modeling analysis, remain limited. It is thus difficult to conduct a comprehensive cost-effectiveness evaluation of SGLT-2i use on all relevant health outcomes (e.g., renal outcomes) or consider all relevant clinical factors in HF patients. Therefore, the present study focused on the most clinically important outcome for HF patients, namely the progression of HF, and applied a well-established HF model to obtain valid study findings. Additionally, given current evidence about the renal protective effect of dapagliflozin, it is expected that the economic benefit of add-on dapagliflozin found in this study, which mainly resulted from the cardiovascular effect of SGLT-2is, would remain or be even more magnified when kidney-related health states are considered in the economic modeling analysis. Nonetheless, the development of a disease model that can comprehensively consider all relevant clinical outcomes or factors in patients with HF is warranted for future research. Seventh, we only included countries with similar healthcare systems (i.e., universal nationwide health insurance coverage) and economic status (i.e., developed countries) in the Asia–Pacific region to avoid potential variations in study findings that may be introduced by different healthcare systems and economic levels. Therefore, the generalizability of our findings to countries with different healthcare systems and economic levels should be done with caution. Lastly, the present study did not compare the economic outcomes associated with different SGLT-2is. Due to the lack of head-to-head comparisons of individual SGLT-2is among HF patients, there may not be sufficient and reliable information as model input parameters (e.g., transition probabilities) for comparative economic analyses of individual SGLT-2is. Future research should explore the variation in the health outcomes and economic costs for HF patients on different SGT-2is.

## Conclusions

This study suggests that add-on dapagliflozin to standard care compared to standard care alone for patients with HFrEF would be cost-effective from the perspective of healthcare systems in Asian-Pacific countries. Future research that considers patients with different characteristics in real-world settings is warranted to promote personalized medicine and optimize health policy decision making.

## Supplementary Information

**Additional file 1**: Overview of detailed model structure of base-case cost-effectiveness analysis.

**Additional file 2**: Overview of detailed model structure of cost-effectiveness analysis where adverse events of treatment were considered.

**Additional file 3** Study parameters associated with hospitalizations for adverse events of treatments in the simulation model (sensitivity analysis).

**Additional file 4**: Cost and utility inputs for cost-effectiveness analysis of add-on dapagliflozin to standard care versus standard care alone in other Asia-Pacific countries.

**Additional file 5**: Scatter plot of distribution of incremental cost and effectiveness ratios under the willingness-to-pay threshold of US$ 25,000 in Taiwan.

**Additional file 6**: Subgroup analyses based on (a) race, (b) age, (c) type 2 diabetes mellitus (T2DM) status, (d) New York Heart Association functional class (NYHA Fc), (e) left ventricular ejection fraction (LVEF), and (f) history of ischemic heart failure (HF).

**Additional file 7:** Probability of cost-effectiveness of adding dapagliflozin to standard care versus standard care alone under different monthly costs of dapagliflozin.

**Additional file 8:** Cost-effectiveness of add-on dapagliflozin to standard care versus standard care alone in Asia-Pacific countries.

**Additional file 9**: Observed and simulated survival curves of patients with stable heart failure.

## Data Availability

All data generated or analyzed during the cost-effectiveness analysis are included in this published article and its supplementary information files.

## Abbreviations

HF: Heart failure
HHF: Hospitalization for heart failure
HFrEF: Heart failure with reduced ejection fraction
SGLT-2i: Sodium-glucose cotransporter-2 inhibitor
DAPA-HF: Dapagliflozin in patients with heart failure and reduced ejection fraction
NYHA Fc: New York Heart Association functional class
NHIRD: National Health Insurance Research Database
QALY: Quality-adjusted life-year
ICER: Incremental cost-effectiveness ratio
WTP: Willingness-to-pay
GDP: Gross domestic product
DSA: Deterministic sensitivity analysis
PSA: Probabilistic sensitivity analysis
LY: Life-year
