# Supplementary material for: Cost-effectiveness evaluation of add-on dapagliflozin for heart failure with reduced ejection fraction from perspective of healthcare systems in Asia–Pacific region
Source: Cardiovasc Diabetol. 2021 Oct 9;20:204. doi: 10.1186/s12933-021-01387-3 (PMC8502298; doi:10.1186/s12933-021-01387-3)
Supplement: Supplementary file 1 — Additional file 1: Overview of detailed model structure of base-case cost-effectiveness analysis. [file 12933_2021_1387_MOESM1_ESM.pdf]

Additional file 1. Overview of detailed model structure of base-case cost-effectiveness analysis

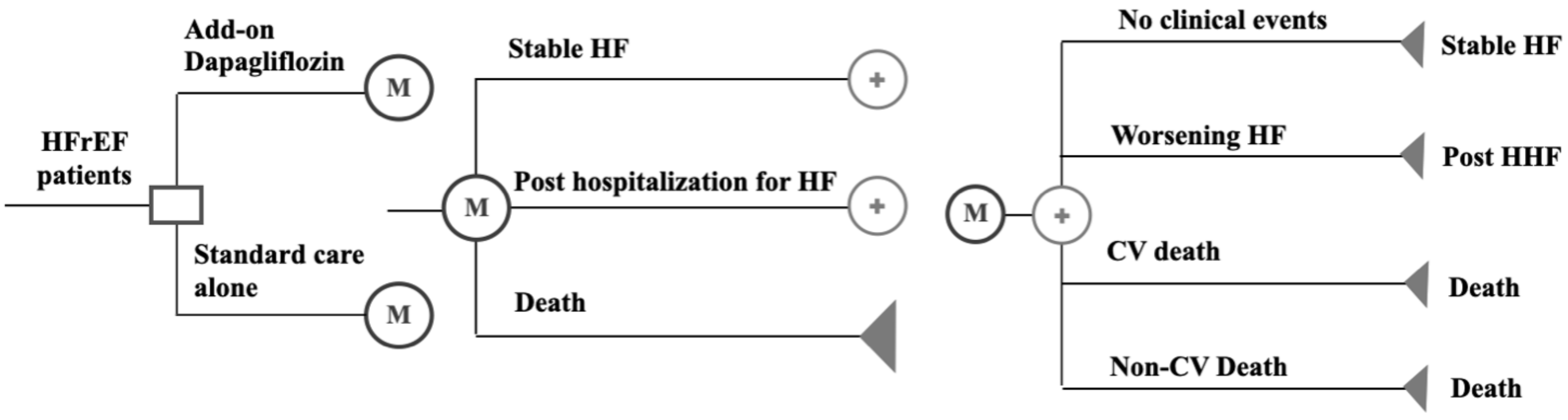

Abbreviations: HFrEF, heart failure with reduced ejection fraction; HF, heart failure; CV, cardiovascular.
