# Supplementary material for: Cost-effectiveness evaluation of add-on dapagliflozin for heart failure with reduced ejection fraction from perspective of healthcare systems in Asia–Pacific region
Source: Cardiovasc Diabetol. 2021 Oct 9;20:204. doi: 10.1186/s12933-021-01387-3 (PMC8502298; doi:10.1186/s12933-021-01387-3)
Supplement: Supplementary file 3 — Additional file 3 Study parameters associated with hospitalizations for adverse events of treatments in the simulation model (sensitivity analysis). [file 12933_2021_1387_MOESM3_ESM.pdf]

Additional file 3. Study parameters associated with hospitalizations for adverse events of treatments in the simulation model (sensitivity analysis)

**Monthly transition probabilities**	**Estimate**	**Standard deviation**	**Distribution**	**Data source**
Hypoglycemia				DAPA-HF trial [10]
Add-on dapagliflozin	0.0000926909	0.000197837	Beta	
Standard care	0.0000928868	0.000198046	Beta	
Diabetic ketoacidosis				
Add-on dapagliflozin	0.0000695043	0.000171317	Beta	
Standard care	0	0	Beta	
Bone fracture				
Add-on dapagliflozin	0.001148223	0.000695941	Beta	
Standard care	0.001171894	0.00070307	Beta	
Amputation				
Add-on dapagliflozin	0.000302426	0.000357317	Beta	
Standard care	0.000279107	0.000343268	Beta	
**Cost parameters (per month)**	**Cost (US$)**	**Range**	**Distribution**	**Data source**
Hypoglycemia	136	68	Gamma	NHIRD
Diabetic ketoacidosis	1,100	550	Gamma	NHIRD
Bone fracture	1,350	675	Gamma	NHIRD
Amputation	7,877	3,938.5	Gamma	NHIRD
**Health utilities parameters**	**Estimate**	**Standard error**	**Distribution**	**Data source**
Hypoglycemia	-0.014*	0.001	Beta	[19]

Diabetic ketoacidosis	-0.037*	0.015	Beta
Bone fracture	-0.148*	0.033	Beta
Amputation	-0.280*	0.053	Beta

*Indicates the decrement in health utility score associated with the health state or the occurrence of clinical events.
