# Supplementary material for: Cost-effectiveness evaluation of add-on dapagliflozin for heart failure with reduced ejection fraction from perspective of healthcare systems in Asia–Pacific region
Source: Cardiovasc Diabetol. 2021 Oct 9;20:204. doi: 10.1186/s12933-021-01387-3 (PMC8502298; doi:10.1186/s12933-021-01387-3)
Supplement: Supplementary file 4 — Additional file 4: Cost and utility inputs for cost-effectiveness analysis of add-on dapagliflozin to standard care versus standard care alone in other Asia-Pacific countries. [file 12933_2021_1387_MOESM4_ESM.pdf]

Additional file 4. Cost and utility inputs for cost-effectiveness analysis of add-on dapagliflozin to standard care versus standard care alone in other Asia-Pacific countries

	**Japan***	**Korea†**	**Singapore‡**	**Australia§**
Utility score of stable heart failure	0.67	0.67	0.72	0.69
Monthly costs of dapagliflozin (US$)	81	20	48	33
Monthly costs for stable heart failure (US$)	115	70	835	267
Costs of hospitalization for heart failure (US$)	10,234	1,217	3,403	8,048
Costs during one month before death (US$)	15,804	1,824	5,378	3,538

References:

*Circulation Journal. 2004;68(1):35-40.

†*Clinical therapeutics.* 2019;41(6):1066-1079.

‡ *Journal of medical economics.* 2018;21(2):174-181.

§*Heart, Lung and Circulation.* 2020;29(9):1310-1317.
