# Supplementary material for: Cost-effectiveness evaluation of add-on dapagliflozin for heart failure with reduced ejection fraction from perspective of healthcare systems in Asia–Pacific region
Source: Cardiovasc Diabetol. 2021 Oct 9;20:204. doi: 10.1186/s12933-021-01387-3 (PMC8502298; doi:10.1186/s12933-021-01387-3)

Additional file 5. Scatter plot of distribution of incremental cost and effectiveness ratios under the willingness-to-pay threshold of US$ 25,000 in Taiwan

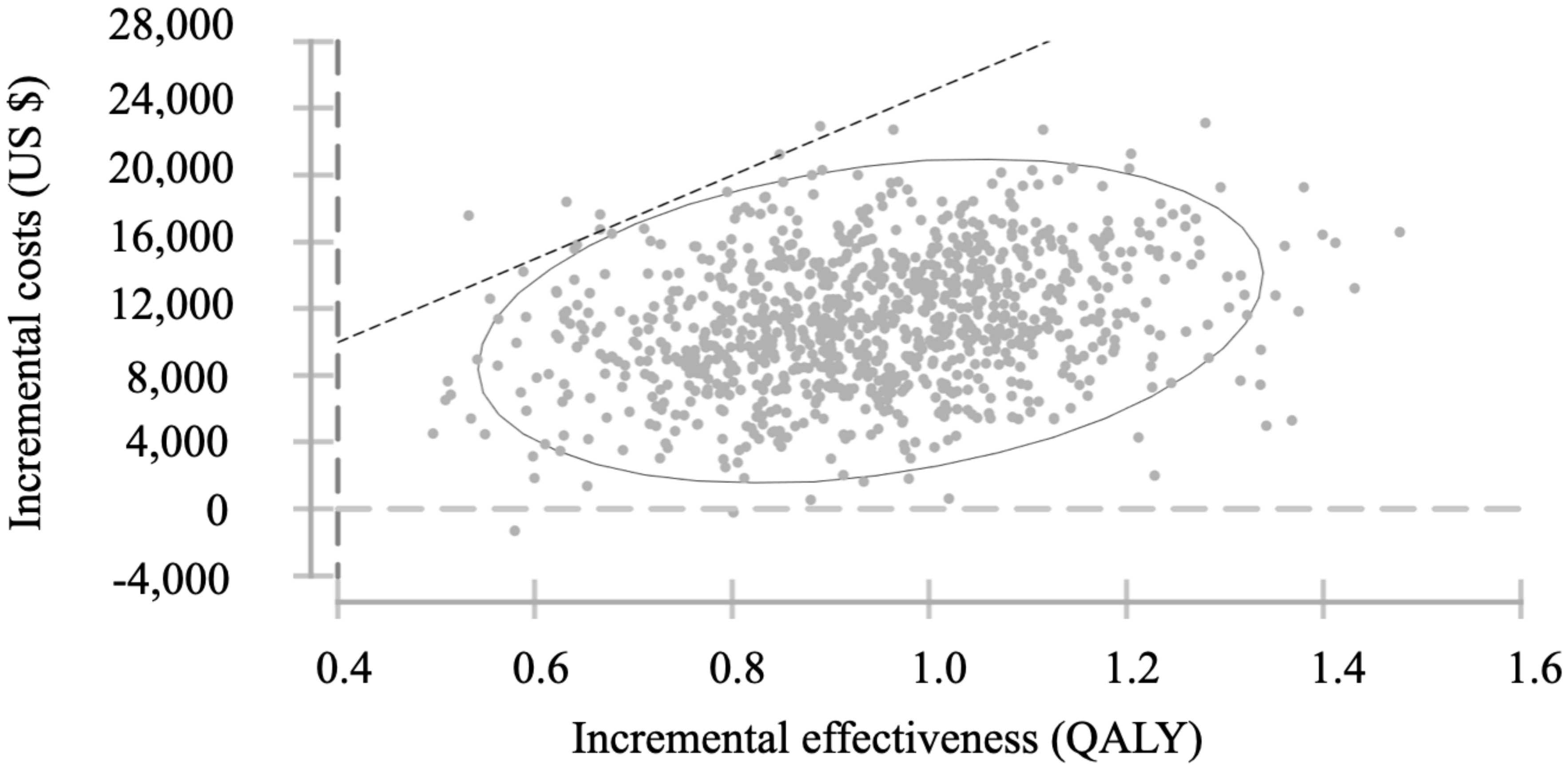

Supplement: Supplementary file 5 — Additional file 5: Scatter plot of distribution of incremental cost and effectiveness ratios under the willingness-to-pay threshold of US$ 25,000 in Taiwan. [file 12933_2021_1387_MOESM5_ESM.pdf]
