# Supplementary material for: Cost-effectiveness evaluation of add-on dapagliflozin for heart failure with reduced ejection fraction from perspective of healthcare systems in Asia–Pacific region
Source: Cardiovasc Diabetol. 2021 Oct 9;20:204. doi: 10.1186/s12933-021-01387-3 (PMC8502298; doi:10.1186/s12933-021-01387-3)

Additional file 6. Subgroup analyses based on (a) race, (b) age, (c) type 2 diabetes mellitus (T2DM) status, (d) New York Heart Association functional class (NYHA Fc), (e) left ventricular ejection fraction (LVEF), and (f) history of ischemic heart failure (HF)

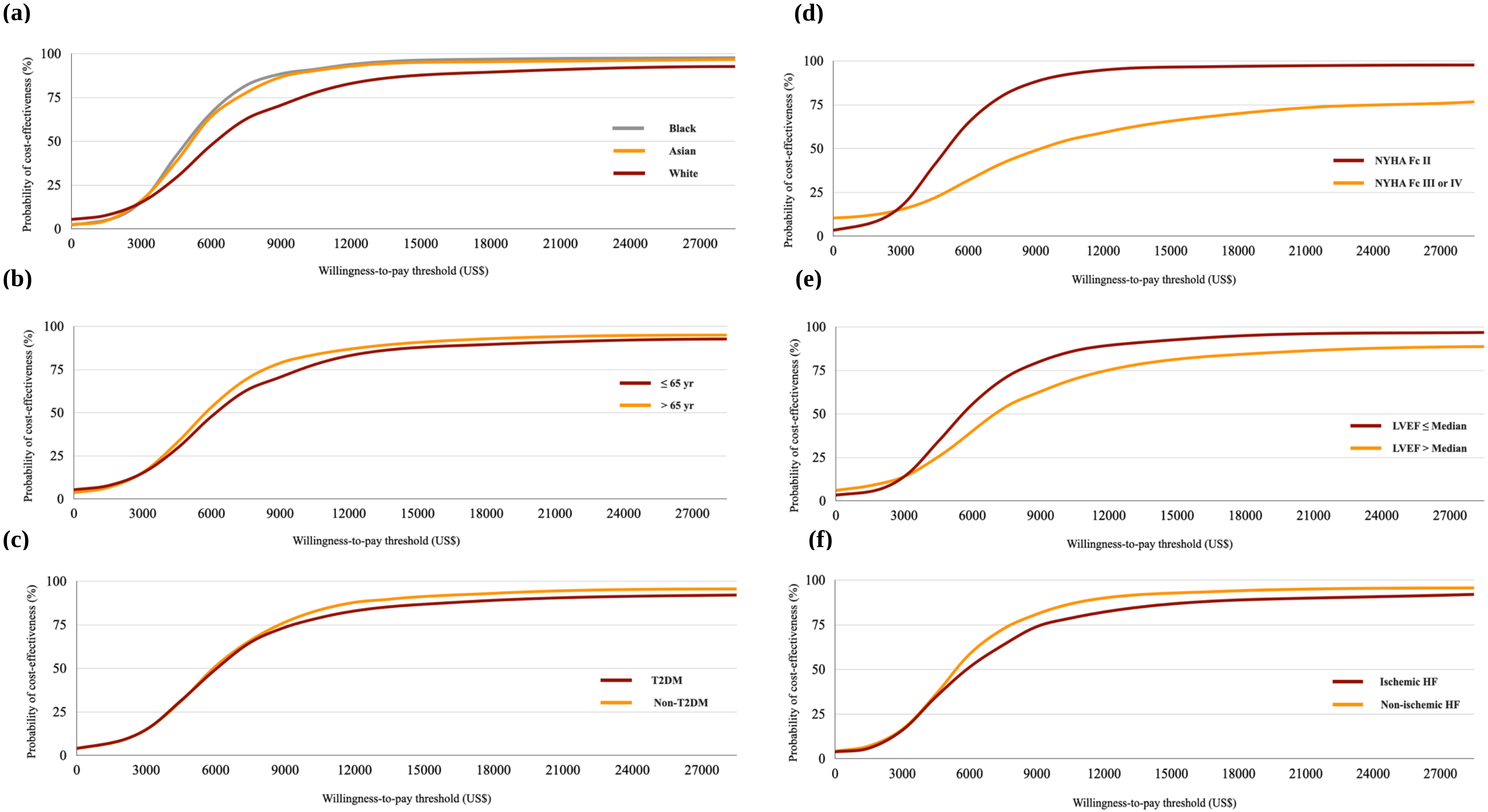

Supplement: Supplementary file 6 — Additional file 6: Subgroup analyses based on (a) race, (b) age, (c) type 2 diabetes mellitus (T2DM) status, (d) New York Heart Association functional class (NYHA Fc), (e) left ventricular ejection fraction (LVEF), and (f) history of ischemic heart failure (HF). [file 12933_2021_1387_MOESM6_ESM.pdf]
