# Supplementary material for: Cost-effectiveness evaluation of add-on dapagliflozin for heart failure with reduced ejection fraction from perspective of healthcare systems in Asia–Pacific region
Source: Cardiovasc Diabetol. 2021 Oct 9;20:204. doi: 10.1186/s12933-021-01387-3 (PMC8502298; doi:10.1186/s12933-021-01387-3)

Additional file 7. Probability of cost-effectiveness of adding dapagliflozin to standard care versus standard care alone under different monthly costs of dapagliflozin

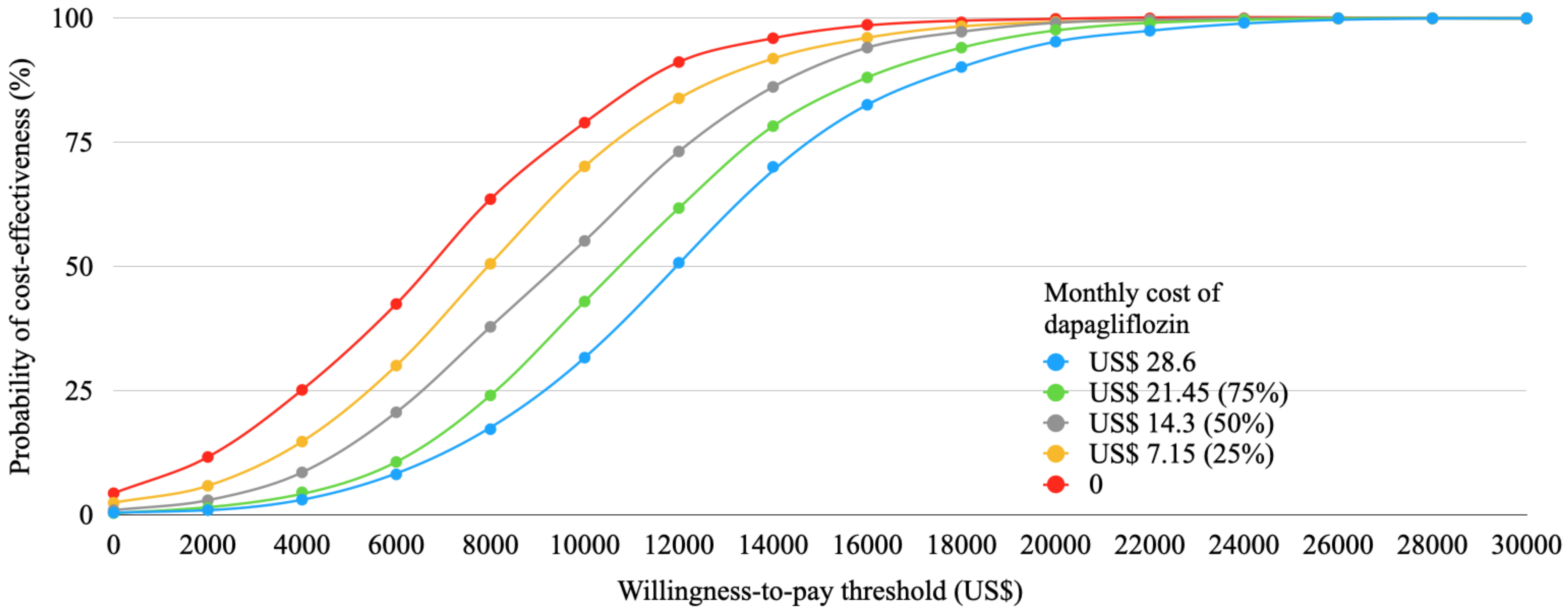

Supplement: Supplementary file 7 — Additional file 7: Probability of cost-effectiveness of adding dapagliflozin to standard care versus standard care alone under different monthly costs of dapagliflozin. [file 12933_2021_1387_MOESM7_ESM.pdf]
