# Supplementary material for: Cost-effectiveness evaluation of add-on dapagliflozin for heart failure with reduced ejection fraction from perspective of healthcare systems in Asia–Pacific region
Source: Cardiovasc Diabetol. 2021 Oct 9;20:204. doi: 10.1186/s12933-021-01387-3 (PMC8502298; doi:10.1186/s12933-021-01387-3)
Supplement: Supplementary file 8 — Additional file 8: Cost-effectiveness of add-on dapagliflozin to standard care versus standard care alone in Asia-Pacific countries. [file 12933_2021_1387_MOESM8_ESM.pdf]

Additional file 8. Cost-effectiveness of add-on dapagliflozin to standard care versus standard care alone in Asia-Pacific countries

	**Cost (US$)**			**QALYs**			**Cost-effectiveness analyses**	
	**Add-on dapagliflozin**	**Standard care**	**ΔCost**	**Add-on dapagliflozin**	**Standard care**	**ΔQALYs**	**ICER (US$)**	**PSA***
Taiwan	87,805	76,501	11,304	11.03	10.09	0.94	12,035	99.3%
Japan	49,064	35,453	13,611	9.56	8.74	0.81	16,705	99.8%
Korea	17,577	13,277	4,300	9.56	8.74	0.81	5,277	100%
Singapore	160,525	140,153	20,372	10.29	9.42	0.88	23,227	99.9%
Australia	59,126	50,745	8,381	9.85	9.01	0.84	9,980	100%

Abbreviations: QALY, quality-adjusted life-year; ICER, incremental cost-effectiveness ration; PSA, probabilistic sensitivity analyses; WTP, willingness to pay.

*The PSA results indicate the probability of cost-effectiveness under 10,000 iterations when add-on dapagliflozin versus standard care alone at the willingness-to-pay threshold of one gross domestic product (GDP) per capita of the given country. The GDP per capita in 2020 was US$ 25,000 for Taiwan, US$ 39,000 for Japan, US$ 30,000 for Korea, US$ 58,000 for Singapore, and US$ 52,000 for Australia.
