# Supplementary material for: Cost-effectiveness evaluation of add-on dapagliflozin for heart failure with reduced ejection fraction from perspective of healthcare systems in Asia–Pacific region
Source: Cardiovasc Diabetol. 2021 Oct 9;20:204. doi: 10.1186/s12933-021-01387-3 (PMC8502298; doi:10.1186/s12933-021-01387-3)
Supplement: Supplementary file 9 — Additional file 9: Observed and simulated survival curves of patients with stable heart failure. [file 12933_2021_1387_MOESM9_ESM.pdf]

Additional file 9. Observed and simulated survival curves of patients with stable heart failure

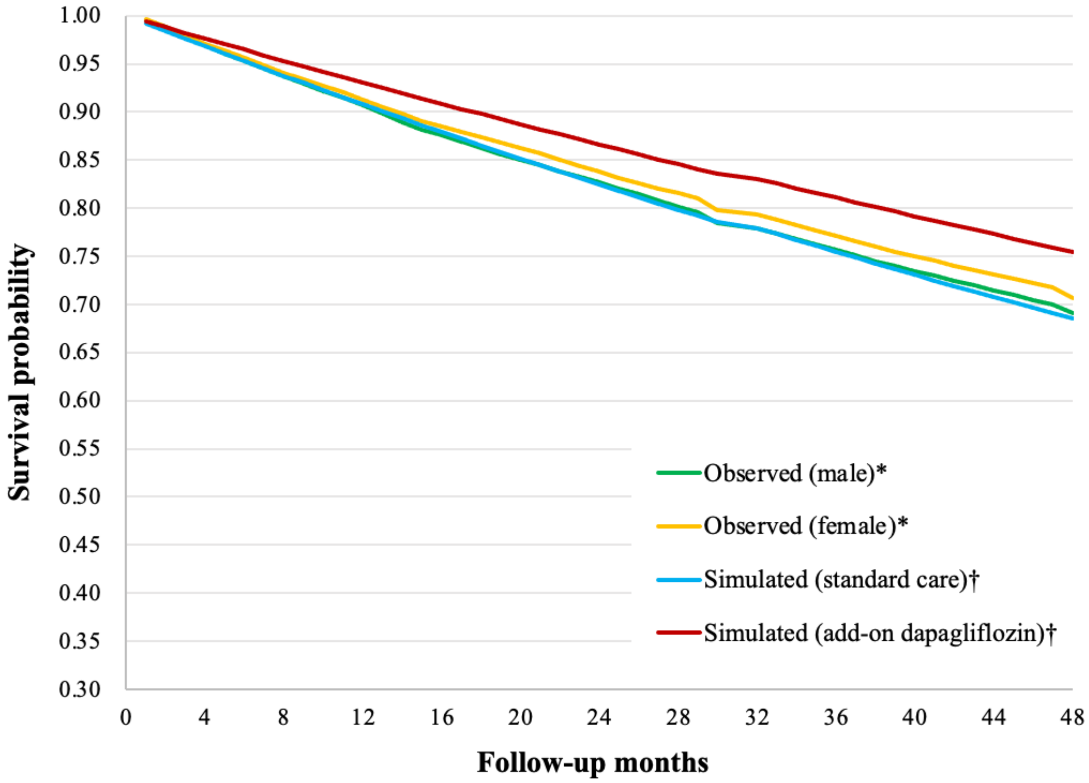

Notes:

*The observed survival curves were obtained from patients with stable heart failure in Taiwan's National Health Insurance Research Database. Detailed cohort selection criteria of patients with stable heart failure are provided in the methods section.

†The simulated survival curves were projected using the heart failure model (Figure 1) with data inputs from the DAPA-HF trial [10].
